# *Homo sapiens*—A Species Not Designed for Space Flight: Health Risks in Low Earth Orbit and Beyond, Including Potential Risks When Traveling beyond the Geomagnetic Field of Earth

**DOI:** 10.3390/life13030757

**Published:** 2023-03-10

**Authors:** David A. Hart

**Affiliations:** Department of Surgery, Faculty of Kinesiology, McCaig Institute for Bone & Joint Health, University of Calgary, Calgary, AB T2N 4N1, Canada; hartd@ucalgary.ca

**Keywords:** *Homo sapiens*, microgravity, low Earth orbit, geomagnetic field, radiation risks, species heterogeneity, adaptation to space, evolution

## Abstract

*Homo sapiens* and their predecessors evolved in the context of the boundary conditions of Earth, including a 1 g gravity and a geomagnetic field (GMF). These variables, plus others, led to complex organisms that evolved under a defined set of conditions and define how humans will respond to space flight, a circumstance that could not have been anticipated by evolution. Over the past ~60 years, space flight and living in low Earth orbit (LEO) have revealed that astronauts are impacted to varying degrees by such new environments. In addition, it has been noted that astronauts are quite heterogeneous in their response patterns, indicating that such variation is either silent if one remained on Earth, or the heterogeneity unknowingly contributes to disease development during aging or in response to insults. With the planned mission to deep space, humans will now be exposed to further risks from radiation when traveling beyond the influence of the GMF, as well as other potential risks that are associated with the actual loss of the GMF on the astronauts, their microbiomes, and growing food sources. Experimental studies with model systems have revealed that hypogravity conditions can influence a variety biological and physiological systems, and thus the loss of the GMF may have unanticipated consequences to astronauts’ systems, such as those that are electrical in nature (i.e., the cardiovascular system and central neural systems). As astronauts have been shown to be heterogeneous in their responses to LEO, they may require personalized countermeasures, while others may not be good candidates for deep-space missions if effective countermeasures cannot be developed for long-duration missions. This review will discuss several of the physiological and neural systems that are affected and how the emerging variables may influence astronaut health and functioning.

## 1. Introduction

### 1.1. Purpose of the Review

For the past >1,000,000 years, humans have evolved under the boundary conditions of Earth. This led to the development of a number of human subspecies, including Neanderthals, which went extinct as a species 30–40,000 years ago. The current dominant subspecies are the *Homo sapiens*, who evolved, presumably in Africa ~200,000 years ago, into the variant that now has gone into space, mainly in low Earth orbit (LEO), plus short trips to the Moon. This experience over the past 60 years of space flight has revealed a number of consequences to living in microgravity in LEO, but still within the context of the geomagnetic field of the Earth. Thus, space flight has revealed many variables that contribute to the biologic set point for *Homo sapiens* living on Earth and the extensive heterogeneity that is embedded in those variables.

Plans are now being developed for extended trips to the Moon and Mars, with the latter being well beyond the influence of the geomagnetic field. The purpose of this review is to discuss the integration of adaptations to living in LEO on physiologic systems, and how going beyond the “protective” effects of the geomagnetic field poses additional risk for the survival and functioning of *Homo sapiens.* Some of these additional risks include the exposure to radiation, with its potential to induce damage to DNA and other molecules, and the potential effects of the loss of the geomagnetic field on neural regulation and the regulation of other physiological systems. The latter area will be a focus of this review, as it is a somewhat understudied area and could potentially have an impact on both astronaut health and the health of future colonists on destinations such as Mars or the Moon.

### 1.2. Background

The Earth has a number of characteristics that are believed to have contributed to the development and evolution of life on the planet. These include, the temperature range, the oxygen tension in an atmosphere, water, nutrients, a 1g gravity, and a geomagnetic field that is believed to protect life from the damaging effects of space radiation and to also protect the integrity of the atmosphere. Various life forms have developed and disappeared over the past millions of years, but they all had to exist and function within the boundary conditions of Earth. Such boundary conditions are not stable, having been subjected to endogenous variations (i.e., ice ages, tectonic plate migration, and atmospheric content), as well as exogenous variables (i.e., asteroid impacts and solar flares). However, the 1 g gravity and the geomagnetic fields are fairly stable in the short term but are not uniform at all sites on the planet. Gravity certainly has influenced many systems [1,2]. How the geomagnetic fields may have impacted evolutionary events indirectly has been advanced by Valkovic [3] and Erdmann et al. [4]. However, whether *Homo sapiens* are dependent on such intrinsic systems that developed during or since the evolution of primitive organisms remains to be elucidated.

Thus, against this backdrop of boundary conditions, plus the thermal, chemical, and nutrient environment, carbon-based lifeforms developed, with many iterations likely arising and disappearing in the past eons. Based on the fossil record that has been obtained thus far, several variations of humanoids have appeared in evolutionary history and then either provided some lineage aspects of *Homo sapiens’* prehistory or became extinct as separate lineage off-shoots. Of course, it is also possible that the evolutionary progression to the current iteration also resulted from interbreeding between the different subspecies, as evidenced by the fact that current *Homo sapiens* retain 1–3% of Neanderthal genes and some *Homo sapiens* also have significant percentages of Denisovan genes [5,6,7]. Why current *Homo sapiens* have retained a subset of Neanderthal and Denisovan genes is not clear, but it sets a precedent for such contributions to contribute to the general heterogeneity in the *Homo sapiens* population. Additional heterogeneity can arise due to a dependence on sex for reproduction, and fetal survival may depend, in part, on histocompatibility gene differences between the female and male [8,9]. All of this background variation, acquired via different mechanisms, could contribute in as yet unknown ways to heterogeneity in the response to humans to space flight and to living in LEO. However, it should be pointed out that genetic studies with astronaut populations that are focused on understanding the variability in response to space conditions have not yet been performed, likely due to the small number of individuals who have been in space for prolonged periods of time (<700 in the past 50 years).

As human evolution could not have anticipated exposure to microgravity during space flight or living in LEO, this heterogeneity in response to microgravity arising during evolution must have remained silent as long as humans stayed on Earth, or the heterogeneity could contribute in some manner to disease development and progression during aging, a time when the integrated physiologic systems may be undergoing senescence and a loss of integrity. Actually, some reports have hypothesized that space flight is a model of aging [10,11,12]. While this analogy between space flight and aging remains to be confirmed and validated, it is clear from the response of humans to space flight that at least one of the boundary conditions of Earth, namely the 1g gravity, was a factor in the design of the upright mobility of humans. If one assumes that this is a precedent for the incorporation of boundary condition variables into the evolutionary development of humans leading to functionality contributing to survival to the present stage of *Homo sapiens*, then one should also likely look for other aspects of these boundary conditions that could impact the integrity of the physiologic systems as humans venture farther and farther from Earth.

Thus, space flight presents both an opportunity to better understand how the boundary conditions of Earth impact human functioning and the potential risks for disease, as well as a set of challenges to overcome if *Homo sapiens* are to become a space-faring species far beyond the confines of Earth. Thus, the solutions to these challenges may also present solutions to the variations within the boundary conditions of the Earth and their risk for disease for those remaining on Earth.

## 2. Responses of Humans to Space Flight and Living in LEO

Humans can be exposed to microgravity for very short durations (i.e., minutes) via parabolic flight in an airplane, and then longer flights in capsules for hours and days, or with the advent of space stations, such as MIR or the International Space Station (ISS), for months to a year. Thus, one can be exposed acutely or chronically to microgravity, with the added exposure to increased radiation on the ISS. With chronic exposure, one may also start to decipher the primary responses and the potential secondary responses due to the complexity of the potential interactions. A number of responses of astronauts to living in LEO conditions have been noted (Table 1); however, the individual responses are quite heterogenous.

### 2.1. Effects on Elements of the Musculoskeletal System (MSK)

The elements of the MSK system, such as the bone, muscle, ligaments, tendons, and menisci, all subscribe to the “use it or lose it” principle on Earth. That is, particularly for bone and muscle, if the tissues are not subjected to biomechanical loading at a level that is specified by the set point, the tissues will undergo atrophy, even on Earth, as reviewed in [13]. On Earth, this can be demonstrated by short- and long-duration bed rest via the removal of an individual from the ground reaction forces (GRF), which are required to continually maintain the integrity of the bone and the muscles, as discussed in [2,11,14]. If one removes a tissue, such as knee menisci, from its in vivo loading environment, it rapidly leads to the induction of a cassette of catabolic genes within 4 h, which can contribute to the atrophy of the tissue [15]. The induction of the catabolic genes can be prevented by intermittent hydrostatic compression in vitro above a threshold [15]. Similarly, one can detect the onset of bone turnover in individuals during bedrest within a few days [16]. Thus, atrophy via catabolism develops when the mechanical loading decreases below a certain level, such as what an astronaut experiences in microgravity.

With the exposure to microgravity on the ISS, there is a fairly rapid induction of bone loss and muscle atrophy, as discussed in [11,12,17]. The bone loss is more from the lower extremities than the upper extremities, likely indicating that the bones of the lower extremities are more exposed to GRF on Earth, and thus, are more likely to be influenced by its loss. The loss of bone is quite variable, with different astronauts losing considerable bone per month (~2%), while others lose much less (~0.1%) per month. There is also a rapid loss of muscle integrity and induction of atrophy [18].

While bone loss can be extensive following exposure to microgravity, there has never been a recorded bone fracture in astronauts while in space. However, bone loss can likely be considered a primary response to microgravity due to the fact that bone requires the gravity-mediated GRF that it evolved to address in response to one of the boundary conditions of Earth. Thus, GRF appears to be central to the regulation of bone, but whether it is the only regulator remains to be confirmed.

However, a secondary consequence of bone loss is the mobilization of the calcium that is liberated from the bone. As calcium is well known to be an important regulator of many enzyme systems and biochemical pathways, the blood levels are tightly regulated and much of the liberated calcium is likely removed from the body by the kidneys and then ends up in the urine. Calcium signaling is important in the heart and vascular systems [19,20,21,22], in the brain [23,24,25], and in other tissues [26,27]. Some individuals who are at risk for kidney stones or gout could suffer from the consequences of high levels of calcium, and some biological systems may also be affected if the removal is not sufficient once the increases in calcium liberation become chronic and a secondary disease risk develops that was not evident on Earth. Therefore, there can be primary, secondary, and potentially tertiary consequences of space-flight-related bone loss.

As bone and muscle are reported to work together as a functional unit [28], and it is well known from studies on Earth that muscles atrophy quickly when they are not used, as reviewed in [11,13,29], it is not surprising that muscle atrophy occurs quickly on exposure to microgravity. As muscles function via neural input at neuromuscular junctions, the loss of muscle integrity in microgravity could be due to the direct effects of loading on muscles and/or the loss of the integrity of the neural component for muscle stimulation.

Whether the other components of the MSK system are directly affected by exposure to microgravity is not well documented. However, it is likely that the tendons may be indirectly affected by muscle atrophy since they are intermediary between the muscles and bone. Many tendons, such as the energy-storing Achilles tendon, function normally on Earth at ~80% of their ultimate stress, and, thus, prolonged muscle atrophy and weakening would lead to the underutilization of tendons and a slower adaptation to this altered state. Whether any adaptations in the tendons would occur primarily in a specific area of the tendon (i.e., enthesis into bone, mid-substance, or the myotendinous junction) remains to be determined.

### 2.2. Countermeasures to Prevent or Reverse Space-Flight Effects on the MSK System

In an attempt to maintain the integrity of the MSK system components, considerable effort has gone into developing countermeasures targeting bone and muscle. These have focused on exercise protocols involving resistance exercises and some aerobic activities. Currently, astronauts on the ISS are supposed to exercise for a few hours/day in order to counteract the effects of microgravity on the MSK system, as reviewed in [18]. While such exercise protocols appear to be capable of helping to retain muscle integrity, they are not as efficient in preventing bone loss [30,31]. Based on such outcomes, either bone and muscle are regulated differently, or the type of exercise that is used is not appropriate to maintain bone integrity. While muscle does appear to respond to exercise in microgravity, in preclinical models, muscle changes can also be attenuated by artificial gravity [32]. Thus, artificial gravity may be a relevant approach to mitigate the effects of microgravity in space. However, in some studies using a bedrest analog, artificial gravity was not effective in mitigating muscle atrophy [33].

If the exercise protocols that are currently in place are not appropriate for bone, what may be more appropriate? While the answer is not known definitively at the present time, there may be clues in what is known about the regulation of bone on Earth. These include the following:

1. Astronauts lose more bone from the lower extremities than the upper extremities, as discussed in [11,12,17];
2. From the work of Frost [34,35,36], bone adapts to mechanical stimulation in response to GRF;
3. The current exercise protocols do not mimic GRF, as GRF loading is likely more of an impact loading than a resistance loading, as discussed in [11,12,17,34,35].

Therefore, perhaps to retain bone requires an impact loading of the lower extremities at the foot to mimic GRF loading. Furthermore, while it is believed that muscle and bone form a functional unit [28], the failure of the current protocols, mainly resistance exercise, to prevent bone loss but allow for the retention of muscle integrity could mean that the current protocols do not allow for fidelity in the functioning of this bone–muscle unit. It is known that active muscles release myokines, which are mediators such as irisin, that can influence other cell types, including bone cells [37]. However, it is also known that some people respond to aerobic exercises and not resistance exercises, and vice versa [38], as discussed in [13]. Perhaps the current protocols do not lead to the release of the appropriate myokines, which can also influence bone and contribute to the effectiveness of an impact loading protocol, or possibly due to sleep disturbances and alterations to circadian rhythms. The released mediators are not effective [39].

While the basis for the differences in the responses of bone and muscle to countermeasures may be due to the countermeasures themselves, the findings thus far may indicate that one should also perhaps look in directions that have not yet been examined in detail. Both bone and muscles are innervated, but in muscle, the functioning is directly related to the extensive network of neuromuscular junctions. In contrast, while bone [40,41,42] and bone marrow [43,44] are innervated, the pattern of innervation of bone indicates that not all cells in the bone are in proximity with nerve endings. Thus, some cells in the bone may play an amplification role regarding the influence of the neural input into the bone environment. Such cells could be the equivalent of the pluripotent regulatory cells that have been postulated to play a role in tissue regulation [45]. In microgravity, such a regulatory mechanism may be compromised, and exercise alone cannot overcome this deficit.

Interestingly, the pharmacological alternative to exercise has also been considered to address the bone loss problem in space [46]. That is, the use of bisphosphonates that are prescribed on Earth for patients with age-related or post-menopausal osteoporosis can also be used in space [46]. Such drugs can be administered as a once-per-year infusion (zoledronic acid) or weekly via the oral route (alendronate and others). While the long-term use of some bisphosphonates does have some side-effects on Earth, such as atypical femoral fractures and osteonecrosis of the jaw, perhaps astronauts will only have to take the drugs in microgravity, and the bone loss on the Moon (1/6 g) or on Mars (1/3 g) will be less. In follow-up studies to those that were reported by Natsu-ume et al. [15], it was found that intermittent loading with 1 or 0.5 MPa completely prevented the induction of the catabolic cassette of genes, while 0.25 MPa was only partially preventative, and 0.1 MPa did not prevent the elaboration of the catabolic genes [Hart et al., unpublished observations]. Thus, in space, a partial g environment may be sufficient to re-establish an intensity of GRF-like loading in order to prevent bone loss. However, in stations such as the planned Gateway on the Moon, astronauts would still be required to take these drugs over an extended period of time.

Interestingly, and relevant to the above discussion, patients with spinal cord injuries (SCI) lose bone below the level of the injury [47], the bone loss is inconsistently attenuated by exercise protocols, and the bone loss does respond, in part, to anti-bone resorptive reagents, such as bisphosphonates and other anti-resorptive reagents such as denosumab, as reviewed in [48]. Thus, there are some interesting parallels between bone loss due to the loss of neural input via SCI and the bone loss after exposure to microgravity. Perhaps these parallels should stimulate some further research to explore a potential neural regulatory basis for bone loss in microgravity environments.

### 2.3. Vascular Alterations in Microgravity and Living in LEO

As astronauts on Earth grow, mature, and function mainly in an upright position, and have tissues that require adequate nutrition and oxygenation, the heart and vascular system has evolved to work effectively against the 1g of Earth in order to maintain system integrity and the ability to remain mobile in such an environment. Therefore, living in a microgravity environment, such as on the ISS or in short-term space flight and long-term space flight, would alleviate the need to work against gravity and require the system set point to adapt to the new conditions. Thus, both acute and chronic adaptations may be evident. Changes to the cardiovascular system have been very evident, and such adaptations have been the subject of considerable investigation [49,50,51,52,53,54,55]. While the adaptations to microgravity have been shown, similar to other response patterns, the response of individual astronauts is variable. In addition, sex differences have been noted in the adaptations and their persistence post-space flight [56,57]. Such alterations can persist for extended periods of time post-flight [58].

As the vascular adaptations to microgravity involve the redistribution of fluids, there can be increased cerebral fluid [53,59] and increased fluid pressures in organs such as the eyes of both astronauts [60,61,62] and preclinical models, such as mice [63]. This can lead to visual disturbances [64], possibly related to effects on the eye itself [65], or the optic nerve [66]. Some of the astronauts have their vision sufficiently altered to require the wearing of glasses.

In order to address the vascular changes, the development of effective countermeasures is needed [67]. The use of artificial gravity has been proposed [68] as a solution to the problem, as well as the use of negative pressure [69,70]. However, this area is in need of more investigation, as the reversibility of the changes may be compromised with increasing the duration of the exposure to microgravity, as well as aging during time in space. However, as will be discussed in later sections, it is likely that effective countermeasures will not be developed for this aspect of space flight in isolation, and that a more holistic perspective on the inter-relationships regarding human adaptations to space flight will be needed. In addition, as there is heterogeneity in the vascular responses to space by astronauts, the basis for such heterogeneity may also provide some clues as to the best way to overcome the vascular consequences of space flight.

Of note, the heart is fundamentally an electrical system that also generates electromagnetic fields. Therefore, it is also potentially affected by magnetic storms that may be encountered in space [71]. As discussed by Baevsky et al. [71], exposure to variations in the geomagnetic field of Earth and magnetic storms are risk factors for cardiovascular disorders. As discussed earlier, calcium ions are also fundamental to the functioning of the heart. In addition, there is an increased exposure of the heart to radiation on the ISS and when living in LEO [72,73,74]. Thus, even in LEO, there are multiple potential stressors that can contribute to the risk of developing cardiovascular disease, such as fluid redistribution due to microgravity, altered calcium regulation, radiation exposure, and magnetic storms. Therefore, developing countermeasures to minimize the impact of the combined and inter-related factors on cardiovascular health will be a challenge, particularly as missions go beyond LEO. Of importance will be the need to make sure that astronauts do not have any underlying subclinical cardiovascular issues prior to missions [75], conditions that could exacerbate the impact of the space environment.

### 2.4. Functional and Structural Brain Changes in LEO and Microgravity

Brain changes at the functional [76,77,78,79] and structural [80] levels can occur during space flight and when living in LEO conditions for long periods of time. Such changes may be the result of fluid shifts and the response to microgravity [81]; however, the space environment contains multiple stressors, including radiation, elevated CO_2_ levels, psychological stress, sleep deprivation, nutritional issues, and others [77,82,83,84,85] that could contribute to alterations in cognition and neural activities. Such changes could compromise the functioning of astronauts during planned deep-space missions [86,87,88].

While some aspects of these space-associated changes in the brain can be captured by analogs such as head-down tilt bedrest studies [89], it is not clear that all of the changes will be detected on Earth, due to the complexity of the changes in space. Interestingly, use of artificial gravity can alleviate some of the neural changes occurring as a result of the bedrest analog condition [90]. In order to mitigate the impact of space-flight-induced changes to brain functioning, will require the development of effective countermeasures [91,92] and tools to detect subtle changes [93], which will require interventions, or biomarker approaches, that could potentially detect the onset of a process that could have a clinical impact [94,95,96]. Whether such functional and structural changes are reversible after chronic exposure in deep space, and whether they are associated with epigenetic alterations, remains to be determined.

### 2.5. Summary of Astronaut Responses to Microgravity and Living in LEO

The response of humans to LEO is complex, with many systems being affected [97,98,99,100] and with a variety of parameters changing in LEO. Some physiological changes are influenced by combinations of factors (Table 1). Based on the above discussion, there are a number of important points that arise regarding human responses to living in LEO for an extended period of time. These include the following:

1. A number of physiologic systems are affected by spending time in LEO. The main stressor appears to be microgravity, but other factors include stress, sleep/circadian rhythm changes, and nutrition, and the affected systems include the musculoskeletal system elements (muscle and bone), the cardiovascular system, the ocular system, and the neural systems, although it is not always clear what are primary effects versus indirect effects;
2. Human responses to LEO are very heterogenous, whether it be the rate of bone loss, cardiovascular adaptations, or functional and structural alterations in the brain. Interestingly, this heterogeneity may be silent on Earth, but could potentially contribute to disease development, particularly in conditions arising during aging;
3. The gut microbiome also appears to be altered during space flight and when living in LEO, a finding that could also lead to alterations in the relationship with the host and that may require separate countermeasures, such as prebiotics [101];
4. Diseases arising during space flight or when living in LEO may not present with the same symptoms and may not respond to interventions the same as on Earth, since the set point for the integrated biological systems would be altered. While not discussed, the immune system of astronauts and animal models is altered during space flight [102,103,104], a factor that may further complicate disease development and intervention efficacy and one that may require specific countermeasures [105];
5. Space flight or living in LEO appears to lead to epigenetic changes in astronauts [106]. While some changes were observed to be reversable once returning to Earth, this may not be the case for all of the changes, and the changes may continue with prolonged time in space;
6. As a number of system stressors arise from living in LEO, the impact on systems such as the brain and cardiovascular systems is complex. Individually, such stressors may evoke responses, but the combined and integrated effect of stressors such as microgravity-mediated fluid redistribution, radiation, calcium regulations, and magnetic storms pose considerable risk to a long-term mission, particularly for those astronauts who may have some underlying subclinical disease or a genetic predilection for disease.

All of the above factors indicate that countermeasures may need to be personalized for each astronaut [100,107], and, furthermore, perhaps not all individuals are genetically and epigenetically suited for space flight. Therefore, selection to minimize the negative impact of space flight in the future may be required, a selection process that would impact the countermeasures and disease risks of individuals.

## 3. Additional Risks of Space Flight into Deep Space and Living on Planets such as Mars

### 3.1. Background

Space flight and living in LEO on facilities such as the ISS exposes astronauts to microgravity and the potential stress of living in close quarters for up to a year; however, these astronauts are still living within the majority of the Earth’s geomagnetic field (GMF). While there is an increased risk for exposure to radiation from the cosmos, the geomagnetic field still exerts a “protective” effect. However, once beyond this protective effect, such as traveling to Mars, astronauts will be beyond the influence of the geomagnetic field and thus exposed to both an increased radiation risk and an environment that has never been experienced by humans for a protracted period of time, namely a lack of being influenced by the geomagnetic field of Earth.

The GMF of Earth is believed to protect the atmosphere of the planet and to protect organisms from the damaging effects of extra-terrestrial radiation from solar flares and the cosmos. Thus, organisms developed replicating systems such as DNA, as well as metabolism products that would be sensitive to damage from radiation if the GMF did not exist. Therefore, the GMF is a boundary condition for the establishment of processes that are essential for life on the planet.

### 3.2. Increased Risk from Space Radiation

As astronauts press deeper into space beyond LEO, there is an increased risk of exposure to different forms of radiation [92,108,109]. Such radiation can result in damage to multiple physiologic systems, resulting in an increased risk for cancer, cardiovascular disease, central nervous system alterations, and others [73,108,109,110]. In addition, for long-term flights with the intent to colonize places such as Mars, radiation could also negatively impact the ovaries and sperm of astronauts [111]. This risk has prompted the investigation of potential countermeasures to diminish or prevent such risks [109].

As with other space-related risks, not all individuals have the same risk for radiation-induced damage [112,113]. Regarding cancer, some individuals have mutations in their suppressor genes, such as BRCA [114], which confer additional risk of cancers. Conversely, there is also polymorphic variation in DNA repair enzymes, which could contribute to disease development after radiation-induced damage [115,116].

Currently, astronauts cannot be discriminated against regarding access to space flight based on their genome. However, as flights become of a longer duration with increased risks, particularly regarding sensitivity to radiation and its consequences, perhaps this restriction should be rescinded, and genomic risk could be factored into suitability for long flights into deep space.

Finally, one should also consider the risks that are posed by radiation on the various microbiomes (gut, skin, and oral) of astronauts. Given the large body of literature regarding the role of the gut microbiome in human health [117,118,119], the impact of space radiation on the development of more virulent organisms, antibiotic resistance, and alterations affecting the microbiota–host interface is of real concern. Certainly, some evidence of environmental radiation impacting the gut microbiome on Earth lends support as to why this aspect of humans in space should be further investigated [120]. This topic has been the subject of a number of recent publications [121,122], including the outcome of a virtual workshop on the topic held in 2020 [123].

In summary, increased radiation risk accompanying space flight to deep space and places such as Mars is of real concern, and to address this risk will require not only improved astronaut selection, but also the development of effective countermeasures to lower the impact of this risk. However, in terms of the risks that are posed by radiation on human health, we also have to consider the effects of radiation on the human microbiomes, particularly the gut microbiota. Interestingly, in mice, the gut microbiota is also influenced by hypogravity [124], therefore, it is likely that there may be interactions between radiation and magnetic fields in alterations to the gut microbiome during space flight.

The question then arises as to what can be undertaken to mitigate the radiation risk. One possibility is the use of medications or drugs that could offer some protection [125]. Certainly, drugs were developed during the cold war to offer protection from nuclear bomb radiation, but the spectrum of radiation is likely different from that encountered in space. Furthermore, in deep-space travel, the exposure will also be chronic, as opposed to acute exposure from bombs on Earth, and Mars having no protective geomagnetic field further complicates taking drugs or medications, potentially for years. Alternatively, one could envision some special shielding for the capsule, possibly an ice shield that could also supply water to the crew. However, due to weight considerations, some shielding may not be practical. This area is definitely in need of continued research and development.

### 3.3. Potential Influence of the GMF Loss on Astronaut Function

The loss of influence by the geomagnetic field of Earth on the functioning and health of astronauts is a relatively understudied area. Given the importance of the GMF of Earth as a significant boundary condition, its role in the evolution of both simple and complex organisms could be anticipated, as reviewed in [126]. Certainly, some birds and other animals use the orientation with the GMF for migration purposes, others use it to orient themselves, and molecules such as transferrin and ferritin use iron in their function [127]. All of these examples indicate that the GMF can be used in some manner in complex organisms. However, it has been more challenging to decipher mechanisms that have developed to deal with the GMF by either incorporating aspects of the GMF into a cellular process, or to negate it. In addition, as both the GMF and gravity are significant boundary conditions, it remains unclear whether some aspects of how simple and complex organisms address these boundary conditions are integrated and/or are completely separate.

Some authors have hypothesized that the magnetic fields that are generated by neural activity could be used to store information [128,129,130,131], such as memory and variables that are related to cognition. For such a system to function properly, it would likely require some intrinsic mechanisms to negate outside influences that are variable (i.e., electromagnetic fields) or accommodate those that are somewhat static (i.e., the GMF of the Earth and local conditions). If it was actually a viable system, it would likely have to accommodate exogenous influences starting in utero. Goult [132] has also proposed that memory has a mechanical basis, a potential mechanism that could be influenced by microgravity. All such hypotheses have not been rigorously tested presently.

An additional complication of modern life that perhaps could not have been anticipated during early evolution is the advent of machinery and equipment that generates electromagnetic fields (EMF) that are used in close proximity to the human body or medical equipment such as magnetic resonance imaging (MRI), which subjects the whole body to an intense magnetic field, but acutely. On Earth, individuals can be exposed to electromagnetic fields in a more chronic manner via devices such as cell phones, household appliances, TVs, computers, or even living close to high-capacity electrical transmission facilities [133]. In space, astronauts are exposed to such EMF from the equipment on the ISS, and in capsules that are headed for deep space, the astronauts will be exposed to EMF continually.

A large number of studies have attempted to better understand the effects of a variety of electromagnetic field intensities over variable periods of time on animals [134,135,136,137,138,139,140], animal tissues [141], and cells [142,143]. The recent review by Lee et al. [140] provides an excellent summary of much of the animal literature in this regard. While many of the studies that were cited examined the gene expression profiles in response to various EMF conditions, the genes affected depended in large part on the animal tissues that were examined or the cells that were used in vitro. Thus, understanding the effects of EMF at the molecular level will be challenging when attempting to translate these results to astronauts.

As the brain and neural activity is fundamentally electrical, considerable investigative effort has been expended to characterize the magnetic fields that are generated by brain activity and how exogenous magnetic fields can affect the brain, as reviewed in [11,12,17,144,145,146,147,148,149]. Several of these approaches require the use of Faraday cages to negate the influence of exogenous electromagnetic field interference. However, Faraday cages would not negate the influence of the GMF, so those brain assessment techniques are assessing function in the context of the GMF. Assessment using such techniques has also raised the issue of the role of magnetic variables in neurodegenerative diseases [150]. In addition, the influence of the orientation and intensity of the local static magnetic field on brain activity [151] also supports a role for the GMF and the local variations in influencing the brain. However, more studies are needed, as this is an understudied area of risk to human health [126].

Additional studies with preclinical model systems have also provided insights into the responses to altered magnetic environments (Table 2). If the development of such magnetic-field-sensitive systems occurred early in evolution, then they should also be evident in less complex organisms and other mammals, but such studies may miss insights into human cognition aspects that could be unique to *Homo sapiens*. The exposure of mammals to hypomagnetic fields (prolonged weakening of the GMF) (Table 2) led to altered immune systems in rats [152], cognitive deficits in the mouse hippocampus [153], and altered noradrenergic activities in the brainstems of hamsters [154]. In Drosophila, exposure to hypomagnetic fields for 10 generations led to amnesia, but this effect was reversible after 6 generations in normal GMF [155]. More recently, it has been reported that exposing Drosophila to the local magnetic field (GMF + local variation) led to imprinting and transgenerational inheritance [156]. Exposure to hypomagnetic fields also led to the altered development of Xenopus embryos [157].

The last point could also be relevant to astronauts in that not all astronauts experienced the same environment for their fetal life and then post-natal growth and maturation. The GMF varies in different parts of the planet (both locally and at the North and South poles versus the equator, and different locations have different concentrations of ferrous metals in the ground). In Western Australia, there are virtual mountains of iron containing taconite and other minerals, and in the Upper Peninsula of Michigan there are towns that were built on high concentrations of hematite (~60–70% iron), as well as taconite (~10% iron). In South Africa (where *Homo sapiens* are believed to have originated and evolved), there are variations in both the GMF, including evidence for pole reversals with accompanying alterations to the GMF [158], and iron ore deposits, which are conditions that could have influenced basal brain regulation in the context of the local magnetic fields during evolution. Therefore, where an astronaut grew up may influence their response to the loss of the GMF, and thus heterogeneity may exist in astronauts with regard to their response to the loss of the GMF. While there are potentially subtle variations while living on Earth, developing in different magnetic conditions could lead to variation in the response patterns to travel to deep space. Unfortunately, to this author’s knowledge, heterogeneity cannot presently be accurately assessed pre-space flight.

While the above discussion has focused on the potential risks that are posed by the loss of the GMF on astronauts, another risk posed by such losses of the GMF could also be felt indirectly via the dependence on growing a food source on a planet such as Mars, or in transit. That is, growing plants in hypogravity has been reported to influence the flowering of *Arabidopsis thaliana* [159], as well as root function [160]. Other plants are also affected by magnetic fields [161], as reviewed in [162]. Therefore, there may be consequences to the loss of influence of the GMF on the food supply for short- and long-term colonies outside of the GMF. However, the relevant plants may adapt to the altered conditions, not unlike the response of Drosophilia [155].

In summary, there is evidence from multiple sources that complex mammals, as well as more simple organisms, generate electromagnetic fields and respond to electromagnetic and static magnetic conditions with alterations to their biological systems, particularly those that are related to brain functioning and cognition. However, it remains somewhat speculative that astronauts will be adversely affected by the loss of the GMF, but there may be heterogeneity in the manner by which astronauts respond to such conditions. Furthermore, the response pattern will likely not be influenced by the loss of the GMF in isolation, as it will also be potentially influenced by microgravity effects on the physiologic systems, the stress of space travel, and living in a confined space for a prolonged period of time. In that context, more effort should be directed to exploring the potential impact of hypomagnetic environments on cognitive functioning and developing approaches to assess the variation in astronauts in this regard.

#### Potential Responses to Loss of the Influence of the GMF of Earth

As discussed above, there is evidence that human brain activity not only generates electromagnetic fields, but the brain can also respond to exogenous magnetic stimulation. Furthermore, magnetic fields can affect various brain functions in animals, such as mice, rats, and hamsters. Finally, eliminating or diminishing the GMF strength can lead to alterations in the activity of brain cells in in vitro studies [163,164,165,166]. The latter includes proliferation, metabolism, and actin and tubulin assembly by human neuroblastoma cells [163,165,166], and the proliferation of mouse neural progenitors and stem cells [164]. Thus, the effects of magnetic fields can be characterized at multiple levels and are not just responses that are associated with the intact human. Based on in vitro studies, one may also conclude that the response of cells to such fields is likely not dependent on iron-dependent events. One additional point that has been made previously that should also be mentioned again is that the development of GMF-related systems arising during the early evolution for simple cells, and potentially later with the onset of more complex organisms, could not have anticipated the ever-expanding impact of electromagnetic fields on complex organisms. This is relevant to the following discussion, as astronauts on space flights to deep space will be surrounded by electromagnetic-field-generating equipment. In part, the response of astronauts to the loss of the GMF of Earth on cognition and other physiologic systems depends on whether the set point is dependent on a passive or active system. A passive system would be designed to merely negate the effect of the GMF, while the active option would be a system actively working against the GMF as part of maintaining the integrity of its function. As humans are heterogeneous, there may be variation in either option, or perhaps the parameters of the set point may make some individuals more sensitive to the EMF than others.

Therefore, given the above discussion, there are a number of alternatives that could happen as humans explore deep space beyond the influence of the GMF of Earth. These include the following:

##### **Option A**—No Effect

As current astronauts have spent their time in utero on Earth, and then grew up on Earth, their GMF-related system set point was determined within the boundary conditions of Earth, dependent, in part, on the local contributions to the ambient magnetic environment. Their exposure to electromagnetic stimuli will also be variable, as will their genetics. Whether chronic exposure to EMF of variable intensities, and with intermittent exposures when living on Earth, causes or results in disease or may predispose individuals to health risks is not known. For >40 years, investigators have examined disease incidence in those living close to power lines, those close to cell phone base stations, cell phone use, and other sources, and the results are inconclusive for the most part [133,167,168,169,170,171,172]. While much of the focus was on EMF effects on children and the development of cancer, the impact on adults and non-cancer effects is negligible when using the variable intensities and durations of the studies. Therefore, for astronauts who are raised on Earth who then go to deep space beyond the GMF of Earth, the impact may be negligible on their cognitive function and other physiological systems, particularly if the set point is based on a passive system that was established during their growth and development.

##### **Option B**—Loss of the GMF has a Significant Impact on Cognition and Cardiovascular Regulation

Clearly, alterations to the brain occur in LEO [89,99,173,174,175], potentially in part due to the microgravity and the associated fluid redistribution that occurs. Therefore, if the GMF-related systems are active, and the brain has already been impacted by microgravity-related changes, then it is likely that the loss of the GMF could exacerbate the changes.

Similarly, the cardiovascular system, including the heart, which is dependent on electrical activity for function, is affected in LEO, and may be further affected by the loss of the GMF. As the heart could also be adversely compromised by radiation, the combined effects could lead to a loss of function during long-term missions to deep space.

An additional variable is the EMF generated by the equipment in the capsule, which then exposes a brain that is no longer “protected” by the GMF to potentially damaging influences. In this circumstance, it may be difficult to attribute specific cause and effect aspects to the loss of the GMF. However, it may be a significant amplifier of changes, leading to functional declines in cognition and other functions, which may compromise the success of missions. This effect could be potentially mitigated by using Faraday cages to protect the astronauts. However, other countermeasures will be needed in order to overcome the direct loss of the GMF, as neural alterations have been noted in early missions.

##### **Option C**—The Lack of a GMF Will Have a Significant Impact on the Offspring of Astronauts Who Are Conceived, Develop, and then Grow in an Environment Lacking a GMF

While it is not an immediate issue, colonists may eventually live on places such as Mars with multigenerational time frames and thus, offspring will be conceived and born in the absence of a GMF in a 1/3 g environment and surrounded by equipment-generated EMF. The 1/3 g may be able to overcome many of the changes occurring in microgravity, but the lack of a GMF during development and early growth and maturation will have never been encountered previously. If the GMF-related systems are passive, it may not matter if a GMF is present. However, if such systems are fundamentally reactive in nature, they will need to develop in reaction to a GMF-equivalent, or many of the brain-related systems may not retain their integrity as they are known on Earth. Furthermore, it may be necessary to shield the living/working quarters, perhaps by using something similar to a Faraday cage, from much of the EMF-generating equipment in order to negate any adverse effects of EMF during development in the absence of a GMF. In addition, devices exerting a static magnetic field may also be useful in substituting for the loss of the GMF. In essence, this may be an experiment that should be performed with animal models in early human colonies. In addition, if the development of memory storage systems is compromised in such models, it may be relevant to the previously discussed theories that have been postulated regarding memory storage in magnetic fields.

These, and other options for the results of living and functioning in conditions lacking a GMF, as well as reduced gravity, an elevated exposure to radiation, and chronic exposure to EMF, present complex challenges for astronaut health. As an organism that has evolved under very controlled boundary conditions, the added risks that are associated with going from LEO conditions to deep space pose challenges that have not been encountered previously, and, while some responses may be speculated upon, for others it will be a real-time encounter, unless the potential of new risks are evaluated using new methods that need to be developed.

## 4. Is It Possible to Assess the Potential Impact of Endogenous Magnetic Fields on Individuals Prior to Deep-Space Travel?

While some studies with plants and preclinical models have indicated that hypomagnetic conditions or environments can influence various biological and physiological processes, such conditions have not been applied to humans, and there could be some ethical issues regarding making the exposure to such conditions a requirement for the selection of astronauts for space travel. However, the impact of acute exposure may not pose as serious of a problem as chronic exposure, and, if acute exposure leads to the elaboration of information indicating a need to further explore the consequences, that would be an important direction for additional research, particularly if any heterogeneity in responsiveness to such conditions was detected.

If heterogeneity in response to hypomagnetic conditions is detected in different individuals on Earth, the basis could have a genetic component, but it also could be derived, in part, from where the individual was conceived, brought to term, and then grew up, as both the geomagnetic field and the endogenous magnetic fields due to local iron concentrations could influence the development of the mechanisms that are involved. Assessing the responsiveness of Inuit people from the far north, people from areas with low background magnetic contributions, and individuals from areas with high levels of iron-containing minerals (i.e., the Upper Peninsula of Michigan, Western Australia, and others) could lead to clues as to how magnetic fields contribute to human characteristics, particularly those that are related to specific brain functions.

While it is admittedly hypothetical and somewhat speculative at this point, investigating such influences of magnetic fields on human development, growth, and maturation while on Earth could lead to disease insights and suitability for deep-space travel. Thus, developing a coherent and integrated research plan to investigate such variables could contribute to multiple areas of outcome.

## 5. Conclusions

Exposure to the conditions of space flight can lead to alterations in a number of physiological systems, as well as systems affecting function, such as those that are related to the brain and cognition. Interestingly, astronauts in LEO are very heterogeneous in regard to their response patterns, indicating that there is variability in the affected systems that is potentially silent if one remained on Earth, although a role for such variation in age-related disease has not been ruled out. With the planning of missions to deep space, with destinations such as Mars, astronauts will be exposed to additional risk factors, such as enhanced radiation doses and the loss of the influence of the geomagnetic field of Earth. The loss of the latter will contribute to the radiation risk, as well as a potential risk to physiological systems and brain functioning. This risk is still potential, as the involvement of the geomagnetic boundary condition of Earth in cognition, brain function, and other physiological systems has been understudied to this point; therefore, how the chronic loss of the GMF will affect human functioning, plus the exposure to EMF from the equipment of the transport vehicle, remains largely unknown. However, in order to prevent unexpected changes to astronaut functioning, as well as to future colonists on destinations such as Mars, this area should be the subject of more intense study prior to undertaking such deep-space missions. This is of particular importance, as not only should the risks be evaluated individually, but they may also interact to combine and form greater risks, as discussed in [176]. Not only will such research help to protect astronauts, but the findings will also provide new and valuable information on the functioning of those remaining on Earth. Finally, genetic contributions to the patterns of responses to space flight stressors (i.e., microgravity, radiation, and magnetic fields) should be investigated with astronauts prior to missions. While the number of astronauts is not large, generating a genetic database will likely lead to insights into the tissue-specific responses to space flight, as well as perhaps more general responses to specific stressors. Some progress has been made in this area with regard to the genetic associations with the development of ophthalmic abnormalities [177], and such approaches should be continued and expanded.

## Figures and Tables

**Table 1:** Summary of Human Responses to Long-Duration Living in LEO ^**a**^.

Tissue/System	Response	Effective Counter Measures	Presumed Cause
**MSK System**			
Muscle	Atrophy	Exercise protocols	Microgravity
Bone	Atrophy	Partial (Exercise/Drugs)	Microgravity
Ligaments	??—Not reported		
Tendons	??—Not reported		
**CV System**			
Heart	Arrhythmia, LV mass, ^**b**^ Cardiac output	Compression, Artificial gravity	Microgravity, Radiation?
Vascular			
*Fluid*	Redistribution	Artificial gravity/Compression	Microgravity
*Tissue*	Altered function	No—most recover on return	Microgravity
	Remodeling	May persist	Microgravity
**Eyes**	Altered vision (fluid and/or neural)	Glasses—some recover on return	Microgravity
**Immune System**	Viral reactivation, White blood cells	Stress reduction	Stress, Circadian disruption
**DNA**	Epigenetic	None—some reversible on return	??—likely multi-causal ^**c**^
**Bone Marrow**	Fat infiltration, Hematopoiesis?	None—recovery on return	Microgravity, Others?
**Neural**			
*Central*	Cognition, Behavior, Working memory, Structural alterations	None presently	Microgravity, Others?
*Peripheral*	Neuromuscular Vestibular Cardiovascular	Exercise	Microgravity

**^a^** This table summarizes many, but not all, of the responses of astronauts to life in LEO conditions. However, individual responses are quite variable, and not all astronauts experience the changes to the same degree. As most astronauts to date have been male, the potential to elucidate sex-specific alterations must await additional flights and tenure of female astronauts at locations such as the ISS. **^b^** Left ventricular mass. **^c^** Multi-causal = stress, circadian rhythm disruption, radiation, alterations to hematopoiesis.

**Table 2:** Influence of Hypomagnetic Conditions on Several Preclinical Model Systems.

Species	Acute/Chronic	Systems Affected	Reference
Rats ^**a**^	Chronic	Immune System	[152]
Mice ^**b**^	Chronic	Cognition/Hippocampus	[153]
Hamsters ^**c**^	Chronic	Noradrenergic/Brain Stem	[154]
Drosophila	Chronic	Amnesia	[155]
Drosophila	Chronic	Behavior	[156]
Xenopus	Chronic	Development/Embryos	[157]

**^a^** Male and female Wistar rats subjected to hypomagnetic fields for 6 months, starting at 2 months of age. **^b^** Male C57BL/6 mice subjected to hypomagnetic fields for 8 weeks. **^c^** Golden hamsters of unreported sex were subjected to hypomagnetic fields for periods of time up to 180 days.

## Data Availability

Not applicable.

## References

[1] Adamopoulos K., Koutsouris D., Zaravinos A., Lambrou G. (2021). Gravitational influence on human living systems and the evolution of species on earth. Molecules.

[2] Hart D.A. (2021). Learning from human responses to deconditioning environments: Improved understanding of the “use it or lose it” principle. Front. Sports Act. Living.

[3] Valkovic V. (1977). A possible mechanism for the influence of geomagnetic field on the evolution of life. Orig. Life.

[4] Erdmann W., Kmita H., Kosicki J.Z., Kaczmarek T. (2021). How the geomagnetic field influences life on earth—An integrated approach to Geomagnetobiology. Orig. Life Evol. Biosph..

[5] Schaefer N.K., Shapiro B., Green R.E. (2021). An ancestral recombination graph of human, neanderthal, and Denisovan genomes. Sci. Adv..

[6] Koller D., Wendt F.R., Pathak G.A., De Lillo A., De Angelis F., Cabrera-Mendoza B., Tucci S., Polimanti R. (2022). Denisovan and neanderthal archaic introgression differentially impacted the genetics of complex traits in modern populations. BMC Biol..

[7] Harvati K., Reyes-Centeno H. (2022). Evolution of homo in the middle and late Pleistocene. J. Hum. Evol..

[8] Beer A.E., Quebbeman J.F. (1986). Immunological mechanisms of survival and “rejection” of the fetal allograft. Mead Johns. Symp. Périnat. Dev. Med..

[9] Reed E., Beer A.E., Hutcherson H., King D.W., Suciu-Foca N. (1991). The alloantibody response of pregnant women and its suppression by soluble HLA antigens and anti-idiotypic antibodies. J. Reprod. Immunol..

[10] Ray E.K. (1991). Introduction: Are aging and space effects similar?. Exp. Gerontol..

[11] Hart D.A. (2019). Influence of space environments in system physiologic and molecular integrity: Redefining the concept of human health beyond the boundary conditions of earth. J. Biomed. Sci. Eng..

[12] Hart D.A. (2019). Potential impact of space environments on developmental and maturational programs which evolved to meet the boundary conditions of earth: Will maturing humans be able to establish a functional biologic system set point under non-earth conditions?. J. Biomed. Sci. Eng..

[13] Hart D.A., Zernicke R.F. (2020). Optimal human functioning requires exercise across the lifespan: Mobility in a 1g environment is intrinsic to the integrity of multiple biological systems. Front. Physiol..

[14] Scott A., Khan K.M., Duronio V., Hart D.A. (2008). Mechanotransduction in human bone: In vitro cellular physiology that under-pins bone changes with exercise. Sports Med..

[15] Natsu-Ume T., Majima T., Reno C., Shrive N.G., Frank C.B., Hart D.A. (2005). Menisci of the rabbit knee require mechanical loading to maintain homeostasis: Cyclic hydrostatic compression in vitro prevents derepression of catabolic genes. J. Orthop. Sci..

[16] Kos O., Hughson R., Hart D., Clément G., Frings-Meuthen P., Linnarsson D., Paloski W., Rittweger J., Wuyts F., Zange J. (2014). Elevated serum soluble CD200 and CD200R as surrogate markers of bone loss under bed rest conditions. Bone.

[17] Hart D.A. (2018). Are we learning as much as possible from spaceflight to better understand health and risks to health on earth, as well as in space?. J. Biomed. Sci. Eng..

[18] Moosavi D., Wolovsky D., Depompeis A., Uher D., Lennington D., Bodden R., Garber C. (2021). The effects of spaceflight microgravity on the musculoskeletal system of humans and animals, with an emphasis on exercise as a countermeasure: A systematic scoping review. Physiol. Res..

[19] Smith K.A., Ayon R.J., Tang H., Makino A., Yuan J.X.-J. (2016). Calcium-sensing receptor regulates cytosolic [Ca^2+^] and plays a major role in the development of pulmonary hypertension. Front. Physiol..

[20] Valentim M.A., Brahmbhatt A.N., Tupling A.R. (2022). Skeletal and cardiac muscle calcium transport regulation in health and disease. Biosci. Rep..

[21] Dixon R.E. (2022). Nanoscale organization, regulation, and dynamic reorganization of cardiac calcium channels. Front. Physiol..

[22] Beghi S., Furmanik M., Jaminon A., Veltrop R., Rapp N., Wichapong K., Bidar E., Buschini A., Schurgers L.J. (2022). Calcium signalling in heart and vessels: Role of calmodulin and downstream calmodulin-dependent protein kinases. Int. J. Mol. Sci..

[23] Gleitze S., Paula-Lima A., Núñez M.T., Hidalgo C. (2021). The calcium-iron connection in ferroptosis-mediated neuronal death. Free. Radic. Biol. Med..

[24] McKinney A.A., Petrova R., Panagiotakos G. (2022). Calcium and activity-dependent signaling in the developing cerebral cortex. Development.

[25] Cimini V., Van Noorden S., Terlizzi C., Altobelli G.G. (2022). Calcium/calmodulin-dependent kinases in the hypothalamus, pituitary, and pineal gland: An overview. Int. J. Endocrinol..

[26] Al-Khannaq M., Lytton J. (2022). Regulation of K^+^-dependent Na^+^/Ca^2+^-exchangers. Int. J. Mol. Sci..

[27] Danish M., Ahmad R. (2023). Functional pleiotropy of calcium binding protein regucalcin in signaling and diseases. Cell Signal..

[28] Bettis T., Kim B.-J., Hamrick M.W. (2018). Impact of muscle atrophy on bone metabolism and bone strength: Implications for muscle-bone crosstalk with aging and disuse. Osteoporos. Int..

[29] Camera D.M., Smiles W.J., Hawley J.A. (2016). Exercise-induced skeletal muscle signaling pathways and human athletic performance. Free. Radic. Biol. Med..

[30] Sibonga J., Matsumoto T., Jones J., Shapiro J., Lang T., Shackelford L., Smith S., Young M., Keyak J., Kohri K. (2019). Resistive exercise in astronauts on prolonged spaceflights provides partial protection against spaceflight-induced bone loss. Bone.

[31] Gabel L., Liphardt A.-M., Hulme P.A., Heer M., Zwart S.R., Sibonga J.D., Smith S.M., Boyd S.K. (2022). Pre-flight exercise and bone metabolism predict unloading-induced bone loss due to spaceflight. Br. J. Sports Med..

[32] Okada R., Fujita S.-I., Suzuki R., Hayashi T., Tsubouchi H., Kato C., Sadaki S., Kanai M., Fuseya S., Inoue Y. (2021). Transcriptome analysis of gravitational effects on mouse skeletal muscles under microgravity and artificial 1 g onboard environment. Sci. Rep..

[33] De Martino E., Hides J., Elliott J.M., Hoggarth M., Zange J., Lindsay K., Debuse D., Winnard A., Beard D., Cook J.A. (2021). Lumbar muscle atrophy and increased relative intramuscular lipid concentration are not mitigated by daily artificial gravity after 60-day head-down tilt bed rest. J. Appl. Physiol..

[34] Frost H.M. (1987). The mechanostat: A proposed pathogenic mechanism of osteoporosis and the bone mass effects of mechanical and nonmechanical agents. Bone Miner..

[35] Frost H.M. (1998). From Wolff’s Law to the mechanostat: A new “face” of physiology *. J. Orthop. Sci..

[36] Frost H.M. (2004). A 2003 update of bone physiology and Wolff’s Law for clinicians. Angle Orthod..

[37] Buccoliero C., Oranger A., Colaianni G., Pignataro P., Zerlotin R., Lovero R., Errede M., Grano M. (2021). The effect of irisin on bone cells in vivo and in vitro. Biochem. Soc. Trans..

[38] Ardavani A., Aziz H., Phillips B.E., Doleman B., Ramzan I., Mozaffar B., Atherton P.J., Idris I. (2021). Indicators of response to exercise training: A systematic review and meta-analysis. BMJ Open.

[39] Hart D.A., Zernicke R.F., Shrive N.G. (2022). *Homo sapiens* may incorporate daily acute cycles of “conditioning-deconditioning” to maintain musculoskeletal integrity: Need to integrate with biological clocks and circadian rhythm mediators. Int. J. Mol. Sci..

[40] Wan Q., Qin W., Ma Y., Shen M., Li J., Zhang Z., Chen J., Tay F.R., Niu L., Jiao K. (2021). Crosstalk between bone and nerves within bone. Adv. Sci..

[41] Gerosa L., Lombardi G. (2021). Bone-to-brain: A round trip in the adaptation to mechanical stimuli. Front. Physiol..

[42] Xu J., Zhang Z., Zhao J., Meyers C.A., Lee S., Qin Q., James A.W. (2022). Interaction between the nervous and skeletal systems. Front. Cell Dev. Biol..

[43] Fielding C., Méndez-Ferrer S. (2020). Neuronal regulation of bone marrow stem cell niches. F1000Research.

[44] Zhang X., Hassan M.G., Scheller E.L. (2021). Neural regulation of bone marrow adipose tissue. Best Pract. Res. Clin. Endocrinol. Metab..

[45] Hart D.A. (2022). One of the primary functions of tissue-resident pluripotent pericytes cells may be to regulate normal organ growth and maturation: Implications for attempts to repair tissues later in life. Int. J. Mol. Sci..

[46] LeBlanc A., Matsumoto T., Jones J., Shapiro J., Lang T., Shackelford L., Smith S.M., Evans H., Spector E., Ploutz-Snyder R. (2013). Bisphosphonates as a supplement to exercise to protect bone during long-duration spaceflight. Osteoporos. Int..

[47] Antoniou G., Benetos I.S., Vlamis J., Pneumaticos S.G. (2022). Bone mineral density post a spinal cord injury: A review of the current literature guidelines. Cureus.

[48] Bauman W.A. (2021). Pharmacological approaches for bone health in persons with spinal cord injury. Curr. Opin. Pharmacol..

[49] Zhang L.-F. (2001). Vascular adaptation to microgravity: What have we learned?. J. Appl. Physiol..

[50] Hughson R.L., Shoemaker J.K. (2004). Vascular health in space. J. Gravit. Physiol..

[51] Zuj K.A., Arbeille P., Shoemaker J.K., Blaber A.P., Greaves D.K., Xu D., Hughson R.L., Zhang L.-F., Hargens A.R., Marshall-Goebel K. (2012). Impaired cerebrovascular autoregulation and reduced CO_2_ reactivity after long duration spaceflight. Am. J. Physiol. Heart Circ. Physiol..

[52] Bimpong-Buta N.-Y., Jirak P., Wernly B., Lichtenauer M., Masyuk M., Muessig J.M., Braun K., Kaya S., Kelm M., Jung C. (2018). Analysis of human microcirculation in weightlessness: Study protocol and pre-study experiments. Clin. Hemorheol. Microcirc..

[53] Iwasaki K., Ogawa Y., Kurazumi T., Imaduddin S.M., Mukai C., Furukawa S., Yanagida R., Kato T., Konishi T., Shinojima A. (2021). Long-duration spaceflight alters estimated intracranial pressure and cerebral blood velocity. J. Physiol..

[54] Taylor C.R., Hanna M., Behnke B.J., Stabley J.N., McCullough D.J., Davis R.T., Ghosh P., Papadopoulos A., Muller-Delp J.M., Delp M.D. (2013). Spaceflight-induced alterations in cerebral artery vasoconstrictor, mechanical, and structural properties: Im-plications for elevated cerebral perfusion and intracranial pressure. FASEB J..

[55] Lee S.M.C., Ribeiro L.C., Martin D.S., Zwart S.R., Feiveson A.H., Laurie S.S., Macias B.R., Crucian B.E., Krieger S., Weber D. (2020). Arterial structure and function during and after long-duration spaceflight. J. Appl. Physiol..

[56] Platts S.H., Merz C.N.B., Barr Y., Fu Q., Gulati M., Hughson R., Levine B.D., Mehran R., Stachenfeld N., Wenger N.K. (2014). Effects of sex and gender on adaptation to space: Cardiovascular alterations. J. Women’s Health.

[57] Hughson R.L., Robertson A.D., Arbeille P., Shoemaker J.K., Rush J.W.E., Fraser K.S., Greaves D.K. (2016). Increased postflight carotid artery stiffness and inflight insulin resistance resulting from 6-mo spaceflight in male and female astronauts. Am. J. Physiol. Heart Circ. Physiol..

[58] Kramer L.A., Hasan K.M., Stenger M.B., Sargsyan A., Laurie S.S., Otto C., Ploutz-Snyder R.J., Marshall-Goebel K., Riascos R.F., Macias B.R. (2020). Intracranial effects of microgravity: A prospective longitudinal MRI study. Radiology.

[59] Jasien J.V., Laurie S.S., Lee S.M.C., Martin D.S., Kemp D.T., Ebert D.J., Ploutz-Snyder R.J., Marshall-Goebel K., Alferova I.V., Sargsyan A.E. (2022). Noninvasive indicators of intracranial pressure before, during, and after long-duration spaceflight. J. Appl. Physiol..

[60] Buckey J.C., Phillips S.D., Anderson A., Chepko A.B., Archambault-Leger V., Gui J., Fellows A.M. (2018). Microgravity-induced ocular changes are related to body weight. Am. J. Physiol. Regul. Integr. Comp. Physiol..

[61] Hughson R.L., Irving E.L. (2021). Spaceflight not an eye-popping experience for astronauts. J. Physiol..

[62] Wostyn P., Gibson C.R., Mader T.H. (2022). The odyssey of the ocular and cerebrospinal fluids during a mission to Mars: The “ocular glymphatic system” under pressure. Eye.

[63] Mao X.W., Nishiyama N.C., Byrum S.D., Stanbouly S., Jones T., Drew A., Sridharan V., Boerma M., Tackett A.J., Zawieja D. (2019). Characterization of mouse ocular response to a 35-day spaceflight mission: Evidence of blood-retinal barrier disruption and ocular adaptations. Sci. Rep..

[64] Khossravi E.A., Hargens A.R. (2021). Visual disturbances during prolonged space missions. Curr. Opin. Ophthalmol..

[65] Huang A.S., Stenger M.B., Macias B.R. (2019). Gravitational influence on intraocular pressure: Implications for spaceflight and disease. J. Glaucoma.

[66] Wåhlin A., Holmlund P., Fellows A.M., Malm J., Buckey J.C., Eklund A. (2020). Optic nerve length before and after spaceflight. Ophthalmology.

[67] Hughson R.L. (2009). Recent findings in cardiovascular physiology with space travel. Respir. Physiol. Neurobiol..

[68] Zhang L.-F. (2013). Region-specific vascular remodeling and its prevention by artificial gravity in weightless environment. Eur. J. Appl. Physiol..

[69] Watkins W., Hargens A.R., Seidl S., Clary E.M., Macias B.R. (2017). Lower-body negative pressure decreases noninvasively measured intracranial pressure and internal jugular vein cross-sectional area during head-down tilt. J. Appl. Physiol..

[70] Harris K.M., Petersen L.G., Weber T. (2020). Reviving lower body negative pressure as a countermeasure to prevent pathological vascular and ocular changes in microgravity. NPJ Microgravity.

[71] Baevsky R., Petrov V., Chernikova A. (1998). Regulation of autonomic nervous system in space and magnetic storms. Adv. Space Res..

[72] Patel S. (2020). The effects of microgravity and space radiation on cardiovascular health: From low-earth orbit and beyond. Int. J. Cardiol. Heart Vasc..

[73] Rikhi R., Samra G., Arustamyan M., Patel J., Zhou L., Bungo B., Moudgil R. (2020). Radiation induced cardiovascular disease: An odyssey of bedside-bench-bedside approach. Life Sci. Space Res..

[74] Davis C.M., Allen A.R., Bowles D.E. (2021). Consequences of space radiation on the brain and cardiovascular system. J. Environ. Sci. Health C Toxicol. Carcinog..

[75] Hamilton D.R., Murray J.D., Ball C.G. (2006). Cardiac health for astronauts: Coronary calcification scores and CRP as criteria for selection and retention. Aviat. Space Environ. Med..

[76] Clément G., Ngo-Anh J.T. (2013). Space physiology II: Adaptation of the central nervous system to space flight—Past, current, and future studies. Eur. J. Appl. Physiol..

[77] Desai R.I., Limoli C.L., Stark C.E., Stark S.M. (2022). Impact of spaceflight stressors on behavior and cognition: A molecular, neurochemical, and neurobiological perspective. Neurosci. Biobehav. Rev..

[78] Marfia G., Navone S.E., Guarnaccia L., Campanella R., Locatelli M., Miozzo M., Perelli P., Della Morte G., Catamo L., Tondo P. (2022). Space flight and central nervous system: Friends or enemies? Challenges and opportunities for neuroscience and neuro-oncology. J. Neurosci. Res..

[79] Salazar A.P., McGregor H.R., Hupfeld K.E., Beltran N.E., Kofman I.S., De Dios Y.E., Riascos R.F., Reuter-Lorenz P.A., Bloomberg J.J., Mulavara A.P. (2022). Changes in working memory brain activity and task-based connectivity after long-duration spaceflight. Cereb. Cortex.

[80] Doroshin A., Jillings S., Jeurissen B., Tomilovskaya E., Pechenkova E., Nosikova I., Rumshiskaya A., Litvinova L., Rukavishnikov I., De Laet C. (2022). Brain connectometry changes in space travelers after long-duration spaceflight. Front. Neural Circuits.

[81] Marušič U., Meeusen R., Pišot R., Kavcic V. (2014). The brain in micro- and hypergravity: The effects of changing gravity on the brain electrocortical activity. Eur. J. Sport Sci..

[82] Zwart S.R., Mulavara A.P., Williams T.J., George K., Smith S.M. (2021). The role of nutrition in space exploration: Implications for sensorimotor, cognition, behavior and the cerebral changes due to the exposure to radiation, altered gravity, and isolation/confinement hazards of spaceflight. Neurosci. Biobehav. Rev..

[83] McGregor H.R., Lee J.K., Mulder E.R., De Dios Y.E., Beltran N.E., Kofman I.S., Bloomberg J.J., Mulavara A.P., Seidler R.D. (2021). Brain connectivity and behavioral changes in a spaceflight analog environment with elevated CO_2_. Neuroimage.

[84] Cekanaviciute E., Rosi S., Costes S.V. (2018). Central nervous system responses to simulated galactic cosmic rays. Int. J. Mol. Sci..

[85] Tays G.D., Hupfeld K.E., McGregor H.R., Salazar A.P., De Dios Y.E., Beltran N.E., Reuter-Lorenz P.A., Kofman I.S., Wood S.J., Bloomberg J.J. (2021). The effects of long duration spaceflight on sensorimotor control and cognition. Front. Neural Circuits.

[86] Roy-O’Reilly M., Mulavara A., Williams T. (2021). A review of alterations to the brain during spaceflight and the potential relevance to crew in long-duration space exploration. NPJ Microgravity.

[87] Clément G.R., Boyle R.D., George K.A., Nelson G.R., Reschke M.F., Williams T.J., Paloski W.H. (2020). Challenges to the central nervous system during human spaceflight missions to Mars. J. Neurophysiol..

[88] Kokhan V.S., Matveeva M.I., Mukhametov A., Shtemberg A.S. (2016). Risk of defeats in the central nervous system during deep space missions. Neurosci. Biobehav. Rev..

[89] Barkaszi I., Ehmann B., Tölgyesi B., Balázs L., Altbäcker A. (2022). Are head-down tilt bedrest studies capturing the true nature of spaceflight-induced cognitive changes? A review. Front. Physiol..

[90] Tays G.D., McGregor H.R., Lee J.K., Beltran N., Kofman I.S., De Dios Y.E., Mulder E., Bloomberg J.J., Mulavara A.P., Wood S.J. (2022). The effects of 30 minutes of artificial gravity on cognitive and sensorimotor performance in a spaceflight analog environment. Front. Neural Circuits.

[91] Seidler R.D., Stern C., Basner M., Stahn A.C., Wuyts F.L., Zu Eulenburg P. (2022). Future research directions to identify risks and mitigation strategies for neurostructural, ocular, and behavioral changes induced by human spaceflight: A NASA-ESA expert group consensus report. Front. Neural Circuits.

[92] Sihver L. (2021). Biological protection in deep space missions. J. Biomed. Phys. Eng..

[93] Moore T.M., Basner M., Nasrini J., Hermosillo E., Kabadi S., Roalf D.R., McGuire S., Ecker A.J., Ruparel K., Port A.M. (2017). Validation of the cognition test battery for spaceflight in a sample of highly educated adults. Aerosp. Med. Hum. Perform..

[94] Malkani S., Chin C.R., Cekanaviciute E., Mortreux M., Okinula H., Tarbier M., Schreurs A.-S., Shirazi-Fard Y., Tahimic C.G., Rodriguez D.N. (2020). Circulating miRNA spaceflight signature reveals targets for countermeasure development. Cell Rep..

[95] Gaines D., Nestorova G.G. (2022). Extracellular vesicles-derived microRNAs expression as biomarkers for neurological radiation injury: Risk assessment for space exploration. Life Sci. Space Res..

[96] Goukassian D., Arakelyan A., Brojakowska A., Bisserier M., Hakobyan S., Hadri L., Rai A.K., Evans A., Sebastian A., Truongcao M. (2022). Space flight associated changes in astronauts’ plasma-derived small extracellular vesicle microRNA: Biomarker identification. Clin. Transl. Med..

[97] Stella A.B., Ajčević M., Furlanis G., Manganotti P. (2020). Neurophysiological adaptations to spaceflight and simulated microgravity. Clin. Neurophysiol..

[98] Kharlamova A., Proshchina A., Gulimova V., Krivova Y., Soldatov P., Saveliev S. (2021). Cerebellar morphology and behavioural correlations of the vestibular function alterations in weightlessness. Neurosci. Biobehav. Rev..

[99] Carriot J., Mackrous I., Cullen K.E. (2021). Challenges to the vestibular system in space: How the brain responds and adapts to microgravity. Front. Neural Circuits.

[100] Hirayama J., Hattori A., Takahashi A., Furusawa Y., Tabuchi Y., Shibata M., Nagamatsu A., Yano S., Maruyama Y., Matsubara H. (2023). Physiological consequences of space flight, including abnormal bone metabolism, space radiation injury, and circadian clock dysregulation: Implications of melatonin use and regulation as a countermeasure. J. Pineal Res..

[101] Turroni S., Magnani M., Kc P., Lesnik P., Vidal H., Heer M. (2020). Gut microbiome and space travelers’ health: State of the art and possible pro/prebiotic strategies for long-term space missions. Front. Physiol..

[102] Dhar S., Kaeley D.K., Kanan M.J., Yildirim-Ayan E. (2021). Mechano-immunomodulation in space: Mechanisms involving microgravity-induced changes in T cells. Life.

[103] Akiyama T., Horie K., Hinoi E., Hiraiwa M., Kato A., Maekawa Y., Takahashi A., Furukawa S. (2020). How does spaceflight affect the acquired immune system?. NPJ Microgravity.

[104] ElGindi M., Sapudom J., Ibrahim I., Al-Sayegh M., Chen W., Garcia-Sabaté A., Teo J. (2021). May the force be with you (or not): The immune system under microgravity. Cells.

[105] Crucian B.E., Makedonas G., Sams C.F., Pierson D.L., Simpson R., Stowe R.P., Smith S.M., Zwart S.R., Krieger S.S., Rooney B. (2020). Countermeasures-based improvements in stress, immune system dysregulation and latent herpesvirus reactivation onboard the international space station—Relevance for deep space missions and terrestrial medicine. Neurosci. Biobehav. Rev..

[106] Garrett-Bakelman F.E., Darshi M., Green S.J., Gur R.C., Lin L., Macias B.R., McKenna M.J., Meydan C., Mishra T., Nasrini J. (2019). The NASA twins study: A multidimensional analysis of a year-long human spaceflight. Science.

[107] Loriè E.P., Baatout S., Choukér A., Buchheim J.-I., Baselet B., Russo C.D., Wotring V., Monici M., Morbidelli L., Gagliardi D. (2021). The future of personalized medicine in space: From observations to countermeasures. Front. Bioeng. Biotechnol..

[108] Sishc B.J., Zawaski J., Saha J., Carnell L.S., Fabre K.M., Elgart S.R. (2022). The need for biological countermeasures to mitigate the risk of space radiation-induced carcinogenesis, cardiovascular disease, and central nervous system deficiencies. Life Sci. Space Res..

[109] Putt K.S., Du Y., Fu H., Zhang Z.-Y. (2022). High-throughput screening strategies for space-based radiation countermeasure discovery. Life Sci. Space Res..

[110] Guo Z., Zhou G., Hu W. (2022). Carcinogenesis induced by space radiation: A systematic review. Neoplasia.

[111] Ahrari K., Omolaoye T.S., Goswami N., Alsuwaidi H., du Plessis S.S. (2022). Effects of space flight on sperm function and integrity: A systematic review. Front. Physiol..

[112] Hall E., Brenner D., Worgul B., Smilenov L. (2005). Genetic susceptibility to radiation. Adv. Space Res..

[113] Cucinotta F.A., Saganti P.B. (2022). Race and ethnic group dependent space radiation cancer risk predictions. Sci. Rep..

[114] Marchetti C., Ataseven B., Cassani C., Sassu C.M., Congedo L., D’Indinosante M., Cappuccio S., Rhiem K., Hahrem E., Cordisco E.L. (2023). Ovarian cancer onset across different BRCA mutation types: A view to a more tailored approach for BRCA mutated patients. Int. J. Gynecol. Cancer.

[115] Kladova O.A., Fedorova O.S., Kuznetsov N.A. (2022). The role of natural polymorphic variants of DNA polymerase β in DNA repair. Int. J. Mol. Sci..

[116] Jia N., Guo C., Nakazawa Y., Heuvel D.V.D., Luijsterburg M.S., Ogi T. (2021). Dealing with transcription-blocking DNA damage: Repair mechanisms, RNA polymerase II processing and human disorders. DNA Repair.

[117] Meyers G.R., Samouda H., Bohn T. (2022). Short chain fatty acid metabolism in relation to gut microbiota and genetic variability. Nutrients.

[118] Huang Z., Liu K., Ma W., Li D., Mo T., Liu Q. (2022). The gut microbiome in human health and disease—Where are we and where are we going? A bibliometric analysis. Front. Microbiol..

[119] Andrioaie I.-M., Duhaniuc A., Nastase E.V., Iancu L.S., Luncă C., Trofin F., Anton-Păduraru D.-T., Dorneanu O.-S. (2022). The role of the gut microbiome in psychiatric disorders. Microorganisms.

[120] Lavrinienko A., Mappes T., Tukalenko E., Mousseau T., Møller A.P., Knight R., Morton J.T., Thompson L.R., Watts P.C. (2018). Environmental radiation alters the gut microbiome of the bank vole Myodes glareolus. ISME J..

[121] Kirkpatrick A.W., Hamilton D.R., McKee J.L., McDonald B., Pelosi P., Ball C.G., Roberts D.J., McBeth P.B., Coccolini F., Ansaloni L. (2020). Do we have the guts to go? The abdominal compartment, intra-abdominal hypertension, the human microbiome and exploration class space missions. Can. J. Surg..

[122] Caswell G., Eshelby B. (2022). Skin microbiome considerations for long haul space flights. Front. Cell Dev. Biol..

[123] LaPelusa M., Donoviel D., Branzini S.E., Carlson P.E., Culler S., Cheema A.K., Kaddurah-Daouk R., Kelly D., de Cremoux I., Knight R. (2021). Microbiome for Mars: Surveying microbiome connections to healthcare with implications for long-duration human spaceflight, virtual workshop, July 13, 2020. Microbiome.

[124] Zhan A., Luo Y., Qin H., Lin W., Tian L. (2022). Hypomagnetic field exposure affecting gut microbiota, reactive oxygen species levels, and colonic cell proliferation in mice. Bioelectromagnetics.

[125] McLaughlin M.F., Donoviel D.B., Jones J.A. (2017). Novel indications for commonly used medications as radiation protectants in spaceflight. Aerosp. Med. Hum. Perform..

[126] Zhang Z., Xue Y., Yang J., Shang P., Yuan X. (2021). Biological effects of hypomagnetic field: Ground-based data for space exploration. Bioelectromagnetics.

[127] van Eijk H.G., de Jong G. (1992). The physiology of iron, transferrin, and ferritin. Biol. Trace Element Res..

[128] McFadden J. (2002). The conscious electromagnetic information (Cemi) field theory. J. Conscious. Stud..

[129] Arturo M., Banaclocha M. (2005). Magnetic storage of information in the human cerebral cortex: A hypothesis for memory. Int. J. Neurosci..

[130] Banaclocha M.A.M., Bókkon I., Banaclocha H.M. (2010). Long-term memory in brain magnetite. Med. Hypotheses.

[131] Pockett S. (2012). The electromagnetic field theory of consciousness. J. Conscious. Stud..

[132] Goult B.T. (2021). The mechanical basis of memory—The Mesh CODE theory. Front. Mol. Neurosci..

[133] Carles C., Esquirol Y., Turuban M., Piel C., Migault L., Pouchieu C., Bouvier G., Fabbro-Peray P., Lebailly P., Baldi I. (2020). Residential proximity to power lines and risk of brain tumor in the general population. Environ. Res..

[134] Kazemi M., Sahraei H., Aliyari H., Tekieh E., Saberi M., Tavacoli H., Meftahi G.H., Ghanaati H., Salehi M., Hajnasrollah M. (2018). Effects of the extremely low frequency electromagnetic fields on NMDA-receptor gene expression and visual working memory in male rhesus macaques. Basic Clin. Neurosci. J..

[135] Ohtani S., Ushiyama A., Maeda M., Wada K., Suzuki Y., Hattori K., Kunugita N., Ishii K. (2019). Global analysis of transcriptional expression in mice exposed to intermediate frequency magnetic fields utilized for wireless power transfer systems. Int. J. Environ. Res. Public Health.

[136] Jeong Y.J., Son Y., Choi H.D., Kim N., Lee Y.S., Ko Y.G., Lee H.J. (2020). Behavioral changes and gene profile alterations after chronic 1,950-MHz radiofrequency exposure: An observation in C57BL/6 mice. Brain Behav..

[137] Lameth J., Arnaud-Cormos D., Lévêque P., Boillée S., Edeline J.-M., Mallat M. (2020). Effects of a single head exposure to GSM-1800 MHz signals on the transcriptome profile in the rat cerebral cortex: Enhanced gene responses under proinflammatory conditions. Neurotox. Res..

[138] Kim J.H., Yu D.-H., Kim H.-J., Huh Y.H., Cho S.-W., Lee J.-K., Kim H.-G., Kim H.R. (2018). Exposure to 835 MHz radiofrequency electromagnetic field induces autophagy in hippocampus but not in brain stem of mice. Toxicol. Ind. Health.

[139] Huegel J., Chan P.Y.W., Weiss S.N., Nuss C.A., Raja H., Waldorff E.I., Zhang N., Ryaby J.T., Soslowsky L.J., Kuntz A.F. (2022). Pulsed electromagnetic field therapy alters early healing in a rat model of rotator cuff injury and repair: Potential mechanisms. J. Orthop. Res..

[140] Lee H.-J., Jin H., Ahn Y.H., Kim N., Pack J.K., Choi H.-D., Lee Y.-S. (2023). Effects of intermediate frequency electromagnetic fields: A review of animal studies. Int. J. Radiat. Biol..

[141] Drzewiecka E.M., Kozlowska W., Paukszto L., Zmijewska A., Wydorski P.J., Jastrzebski J.P., Franczak A. (2021). Effect of the Electromagnetic Field (EMF) radiation on transcriptomic profile of pig myometrium during the peri-implantation period—An in vitro study. Int. J. Mol. Sci..

[142] Mustafa E., Makinistian L., Luukkonen J., Juutilainen J., Naarala J. (2022). Do 50/60 Hz magnetic fields influence oxidative or DNA damage responses in human SH-SY5Y neuroblastoma cells?. Int. J. Radiat. Biol..

[143] Dittmann K.H., Mayer C., Stephan H., Mieth C., Bonin M., Lechmann B., Rodemann H.P. (2022). Exposure of primary osteoblasts to combined magnetic and electric fields induced spatiotemporal endochondral ossification characteristic gene- and protein expression profiles. J. Exp. Orthop..

[144] Ahadzi G.M., Liston A.D., Bayford R.H., Holder D.S. (2004). Neuromagnetic field strength outside the human head due to impedance changes from neuronal depolarization. Physiol. Meas..

[145] Seki Y., Miyashita T., Kandori A., Maki A., Koizumi H. (2012). Simultaneous measurement of neuronal activity and cortical hemodynamics by unshielded magnetoencephalography and near-infrared spectroscopy. J. Biomed. Opt..

[146] Cao F., An N., Xu W., Wang W., Yang Y., Xiang M., Gao Y., Ning X. (2021). Co-registration comparison of on-scalp magnetoencephalography and magnetic resonance imaging. Front. Neurosci..

[147] Rea M., Holmes N., Hill R.M., Boto E., Leggett J., Edwards L.J., Woolger D., Dawson E., Shah V., Osborne J. (2021). Precision magnetic field modelling and control for wearable magnetoencephalography. Neuroimage.

[148] Petrosino N.J., Cosmo C., Berlow Y.A., Zandvakili A., Wout-Frank M.V., Philip N.S. (2021). Transcranial magnetic stimulation for post-traumatic stress disorder. Ther. Adv. Psychopharmacol..

[149] Kasprzycka W., Naurecka M.L., Sierakowski B.M., Putko P., Mierczyk Z., Chabik G., Dec S., Gaździński S., Rola R. (2022). Recognition and processing of visual information after neuronavigated transcranial magnetic stimulation session. Brain Sci..

[150] Kletetschka G., Bazala R., Takáč M., Svecova E. (2022). Magnetic domains oscillation in the brain with neurodegenerative disease. Sci. Rep..

[151] Wang C.X., Hilburn I.A., Wu D.-A., Mizuhara Y., Cousté C.P., Abrahams J.N.H., Bernstein S.E., Matani A., Shimojo S., Kirschvink J.L. (2019). Transduction of the geomagnetic field as evidenced from alpha-band activity in the human brain. Eneuro.

[152] Roman A., Tombarkiewicz B. (2009). Prolonged weakening of the geomagnetic field (GMF) affects the immune system of rats. Bioelectromagnetics.

[153] Tian L., Luo Y., Zhan A., Ren J., Qin H., Pan Y. (2022). Hypomagnetic field induces the production of reactive oxygen species and cognitive deficits in mice hippocampus. Int. J. Mol. Sci..

[154] Zhang X., Li J.-F., Wu Q.-J., Li B., Jiang J.-C. (2007). Effects of hypomagnetic field on noradrenergic activities in the brainstem of golden hamster. Bioelectromagnetics.

[155] Zhang B., Lu H., Xi W., Zhou X., Xu S., Zhang K., Jiang J., Li Y., Guo A. (2004). Exposure to hypomagnetic field space for multiple generations causes amnesia in Drosophila melanogaster. Neurosci. Lett..

[156] Oh I.-T., Kwon H.-J., Kim S.-C., Kim H.-J., Lohmann K.J., Chae K.-S. (2019). Behavioral evidence for geomagnetic imprinting and transgenerational inheritance in fruit flies. Proc. Natl. Acad. Sci. USA.

[157] Mo W.C., Liu Y., Cooper H.M., He R.Q. (2012). Altered development of Xenopus embryos in a hypomagnetic field. Bioelectromagnetics.

[158] Herries A.I., Shaw J. (2011). Palaeomagnetic analysis of the Sterkfontein palaeocave deposits: Implications for the age of the hominin fossils and stone tool industries. J. Hum. Evol..

[159] Agliassa C., Narayana R., Bertea C.M., Rodgers C.T., Maffei M.E. (2018). Reduction of the geomagnetic field delays *Arabidopsis thaliana* flowering time through downregulation of flowering-related genes. Bioelectromagnetics.

[160] Narayana R., Fliegmann J., Paponov I., Maffei M.E. (2018). Reduction of geomagnetic field (GMF) to near null magnetic field (NNMF) affects Arabidopsis thaliana root mineral nutrition. Life Sci. Space Res..

[161] Shabrangy A., Ghatak A., Zhang S., Priller A., Chaturvedi P., Weckwerth W. (2021). Magnetic field induced changes in the shoot and root proteome of barley (*Hordeum vulgare* L.). Front. Plant Sci..

[162] Hafeez M.B., Zahra N., Ahmad N., Shi Z., Raza A., Wang X., Li J. (2022). Growth, physiological, biochemical and molecular changes in plants induced by magnetic fields: A review. Plant Biol..

[163] Wang D.L., Wang X.S., Xiao R., Liu Y., He R.Q. (2008). Tubulin assembly is disordered in a hypogeomagnetic field. Biochem. Biophys. Res. Commun..

[164] Fu J.-P., Mo W.-C., Liu Y., Bartlett P.F., He R.-Q. (2016). Elimination of the geomagnetic field stimulates the proliferation of mouse neural progenitor and stem cells. Protein Cell.

[165] Mo W.-C., Zhang Z.-J., Wang D.-L., Liu Y., Bartlett P.F., He R.-Q. (2016). Shielding of the geomagnetic field alters actin assembly and inhibits cell motility in human neuroblastoma cells. Sci. Rep..

[166] Wang G.-M., Fu J.-P., Mo W.-C., Zhang H.-T., Liu Y., He R.-Q. (2022). Shielded geomagnetic field accelerates glucose consumption in human neuroblastoma cells by promoting anaerobic glycolysis. Biochem. Biophys. Res. Commun..

[167] Feychting M., Ahlbom A. (1993). Magnetic fields and cancer in children residing near Swedish high-voltage power lines. Am. J. Epidemiol..

[168] Repacholi M. (2012). Concern that “EMF” magnetic fields from power lines cause cancer. Sci. Total. Environ..

[169] Amoon A.T., Swanson J., Magnani C., Johansen C., Kheifets L. (2022). Pooled analysis of recent studies of magnetic fields and childhood leukemia. Environ. Res..

[170] Brabant C., Geerinck A., Beaudart C., Tirelli E., Geuzaine C., Bruyere O. (2022). Exposure to magnetic fields and childhood leukemia: A systematic review and meat-analysis of cas-control and cohort studies. Rev. Environ. Health.

[171] Balmori A. (2022). Evidence for a health risk by RF on humans living around mobile phone base stations: From radiofrequency sickness to cancer. Environ. Res..

[172] Castaño-Vinyals G., Sadetzki S., Vermeulen R., Momoli F., Kundi M., Merletti F., Maslanyj M., Calderon C., Wiart J., Lee A.-K. (2022). Wireless phone use in childhood and adolescence and neuroepithelial brain tumours: Results from the international MOBI-Kids study. Environ. Int..

[173] Stahn A.C., Kühn S. (2022). Extreme environments for understanding brain and cognition. Trends Cogn. Sci..

[174] Shirah B.H., Ibrahim B.M., Aladdin Y., Sen J. (2022). Space neuroscience: Current understanding and future research. Neurol. Sci..

[175] Mhatre S.D., Iyer J., Puukila S., Paul A.M., Tahimic C.G., Rubinstein L., Lowe M., Alwood J.S., Sowa M.B., Bhattacharya S. (2022). Neuro-consequences of the spaceflight environment. Neurosci. Biobehav. Rev..

[176] Mo W., Liu Y., He R. (2014). Hypomagnetic field, an ignorable environmental factor in space?. Sci. China Life Sci..

[177] Smith S.M., Zwart S.R. (2018). Spaceflight-related ocular changes: The potential role of genetics, and the potential of B vitamins as a countermeasure. Curr. Opin. Clin. Nutr. Metab. Care.

